# Allosteric interactions between receptor site 3 and 4 of voltage-gated sodium channels: a novel perspective for the underlying mechanism of scorpion sting-induced pain

**DOI:** 10.1186/s40409-015-0043-6

**Published:** 2015-10-19

**Authors:** Yi-Jun Feng, Qi Feng, Jie Tao, Rong Zhao, Yong-Hua Ji

**Affiliations:** Laboratory of Neuropharmacology and Neurotoxicology, Shanghai University, Nanchen Road 333, Shanghai, 200444 China; Department of Nephrology, Putuo Hospital, Shanghai University of Traditional Chinese Medicine, 164 Lanxi Road, Shanghai, 200062 China; Department of Pharmacology, School of Pharmacy, Fudan University, 826 Zhangheng Road, Shanghai, 201203 China; Department of Neuroscience, Baylor College of Medicine, One Baylor Plaza, Houston, TX 77030 USA

**Keywords:** BmK I, BmK IT2, Synergistic effect, Allosteric interactions

## Abstract

**Background:**

BmK I, a site-3-specific modulator of voltage-gated sodium channels (VGSCs), causes pain and hyperalgesia in rats, while BmK IT2, a site-4-specific modulator of VGSCs, suppresses pain-related responses. A stronger pain-related effect has been previously attributed to *Buthus martensi* Karsch (BmK) venom, which points out the joint pharmacological effect in the crude venom.

**Methods:**

In order to detect the joint effect of BmK I and BmK IT2 on ND7-23 cells, the membrane current was measured by whole cell recording. BmK I and BmK IT2 were applied successively and jointly, and the synergistic modulations of VGSCs on ND7-23 cells were detected.

**Results:**

Larger peak I_Na_ and more negative half-activation voltage were elicited by joint application of BmK I and BmK IT2 than by application of BmK I or BmK IT2 alone. Compared to the control, co-applied BmK I and BmK IT2 also significantly prolonged the time constant of inactivation.

**Conclusions:**

Our results indicated that site-4 toxin (BmK IT2) could enhance the pharmacological effect induced by site-3 toxin (BmK I), suggesting a stronger effect elicited by both toxins that alone usually exhibit opposite pharmacological effects, which is related to the allosteric interaction between receptor site 3 and site 4. Meanwhile, these results may bring a novel perspective for exploring the underlying mechanisms of scorpion sting-induced pain.

## Background

Voltage-gated sodium channels (VGSCs) in most excitable cells are involved in generation of action potentials and closely related to a variety of disorders, including epilepsy and myotonia [1]. Drugs and toxins are proved to interact with multiple regions of VGSCs by affecting their gating properties. So far, at least six different receptor sites are confirmed as pharmacological targets [2].

Scorpion toxins modulating VGSCs are divided into α and β classes according to their distinct binding sites [3, 4]. The Asian scorpion *Buthus martensi* Karsch (BmK) is widely distributed from northwestern China to Mongolia and Korea and its sting can deliver fierce pain [5]. The venom of BmK contains many long-chain neuropeptides. BmK I (a main lethal component), classified as an α-toxin, can overexcite neurons via inhibiting the inactivation of VGSCs upon binding to receptor site 3. Moreover, BmK I may induce spontaneous pain and hyperalgesia after intraplantar injections in rats [6–8]. Another component, BmK IT2, classified as a depressant β-toxin, acts on receptor site 4 to inhibit peak current and shift the voltage-dependent activation to more negative membrane potentials, and then leads to inhibition of neuronal excitability and suppressive effects on nociceptive behavior induced by formalin [9–11].

Notably, these two components that exhibit opposite pharmacological effects coexist in the same venom system during a long evolutionary history. A stronger pain-related behavior has been previously recognized in BmK venom (containing BmK I and BmK IT2) and not in BmK I alone [6]. It remains unclear why BmK IT2 did not suppress, but enhanced pain-related behavior induced by BmK I. In our present study, BmK I and BmK IT2 are co-applied to DRG-type cell line ND7-23 to study their synergistic pharmacological effects by whole cell recording. Our present study aims to provide evidence for synergistic effects of the scorpion toxins through allosteric interaction among different receptor sites. It could give us hints to understand the mechanism underlying the joint pharmacological effect and explain how scorpion stings cause pain.

## Methods

### Solutions and drugs

In the patch-clamp recordings, pipette solution contained (mM): 120 CsF, 10 HEPES, 10 EGTA, 15 NaCl (pH 7.25 with CsOH). Bath solution contained (mM): 140 NaCl, 5 HEPES, 1.3 MgCl_2_, 1 CaCl_2_, 11 glucose, 4.7 KCl (pH 7.4 with NaOH). The pipette and bath solutions were adjusted to osmolarities of 285–290 and 295–300 mOsm, respectively.

BmK I and Bmk IT2 were purified from BmK venom by RP-HPLC according to a method previously described [12, 13]. The purity of isolated toxins was checked by mass spectrometry analysis as well as peptide sequencing, and only those with purity above 99 % were used. In order to prevent adherence of the toxin to the vials and the perfusion apparatus, the toxin was dissolved in the bath solution and supplemented with 1 mg/mL bovine serum albumin (BSA). BSA (1 mg/mL) alone does not alter channel function of ND7-23 cells. Unless otherwise stated, all reagents and drugs were purchased from Sigma-Aldrich.

### Cell culture and whole-cell patch clamp recordings

ND7-23 cells were obtained from the Shanghai cell bank of the Chinese Academy of Science (Shanghai, China). The cells were cultured in Dulbecco’s modified Eagle medium (DMEM; Gibco, Invitrogen, USA) and then supplemented with 2 mM l-glutamine, 10 % heat-inactivated fetal bovine serum (FBS; Gibco, Invitrogen). Culture dishes were incubated at 37 °C in a humidified atmosphere containing 5 % CO_2_ and subcultured approximately every 2 to 3 days.

Whole-cell voltage-clamp experiments were performed at room temperature (21-25 °C). Patch pipettes were fabricated from glass capillary tubes by PC-10 Puller (Narishige, Japan) with the resistance of 2–3 MΩ.

Currents were amplified with an Axon 200B patch-clamp amplifer (Axon Instruments, USA) and current signal entered into the computer through AD/DA converter and sampled by Axon pClamp 8.2 software with 20 KHz sampling frequency. Current signal low-pass filtered at 2 kHz of –3 dB through patch clamp amplifier. Series resistance (Rs) was compensated (85 ~ 90 %) to minimize voltage errors, and leak subtraction was performed by a P/4 protocol.

Steady-state activation: the holding potentials were −120 mV. A 50-ms step of depolarization was applied to elicited sodium current (I_Na_). The maximal inward current from 1 to 10 ms was defined as the amplitude of transient sodium current (I_NaT_). I_Na_ was normalized to the peak I_NaT_. The sodium conductance (G) was calculated using the formula:

$$ \mathrm{G}\left(\mathrm{V}\right) = \kern0.5em \frac{\mathrm{I}\left(\mathrm{V}\right)}{\mathrm{V}\ \hbox{--}\ {\mathrm{E}}_{\mathrm{rev}}} $$

Where V is voltage values, and G(V) and I(V) are stand for the conductance G and the current I at the command voltage V, E_rev_ is the reversal potential of sodium flow estimated from the I–V curve. The conductances were normalized to the maximal value between −90 and +40 mV and fitted to a Boltzmann equation:

$$ \mathrm{f}\left(\mathrm{V}\right) = \kern0.5em \frac{\hbox{--} 1}{1 + \exp \left[\left(\mathrm{V}\ \hbox{--}\ {\mathrm{V}}_{1/2\hbox{-} \mathrm{a}}\right)/{\mathrm{K}}_{\mathrm{a}}\right]}\kern0.5em +\kern0.5em 1 $$

Where V_1/2-a_ is the voltage at which half-maximal activation occurs, and K_a_ describes the slope of the fit.

Steady-state inactivation: the voltage dependence of steady-state inactivation was analyzed by two-pulse protocols, composed of a 400 ms prepulse, potentials ranging from −140 to 0 mV with the increments of 10 mV followed by a test pulse of 0 mV for 80 ms respectively. The amplitudes of the I_Na_ were normalized to their maximal value and plotted as channel availability compared with prepulse potential. Data were then fitted to a Boltzmann equation:

$$ \mathrm{f}\left(\mathrm{V}\right) = \kern0.5em \frac{\left(1\ \hbox{--}\ \mathrm{C}\right)}{1 + \exp \left[\left(\mathrm{V}\ \hbox{--}\ {\mathrm{V}}_{1/2\hbox{-} \mathrm{i}}\right)/{\mathrm{K}}_{\mathrm{i}}\right]}\kern0.5em +\kern0.5em \mathrm{C} $$

Where V_1/2-i_ is the voltage at which 50 % of the inactivation component is inactivated, K_i_ is the slope factor, and C is the steady-state asymptote.

Inactivation kinetics: the inactivation kinetics was analyzed by fitting the decay course of I_NaT_ to a single exponential function:

$$ \mathrm{I} = \kern0.5em \mathrm{A}* \exp\ \left(\hbox{--} \mathrm{t}\ /\uptau \right)\kern0.5em  + {\mathrm{I}}_{\mathrm{SS}} $$

Where τ is the recovery time constant.

### Data analysis

The raw data were analyzed by Clampfit 8.2 and Origin 8.5. The results were shown as means ± SEM with the number of experiments shown in the figure legends. Differences between means were analyzed by Student’s test or by one-way ANOVA, *p* < 0.05 was considered a significant difference.

## Results

### Enhanced peak current of sodium channels in ND7-23 after co-application of BmK I and BmK IT2

**Fig. 1:**
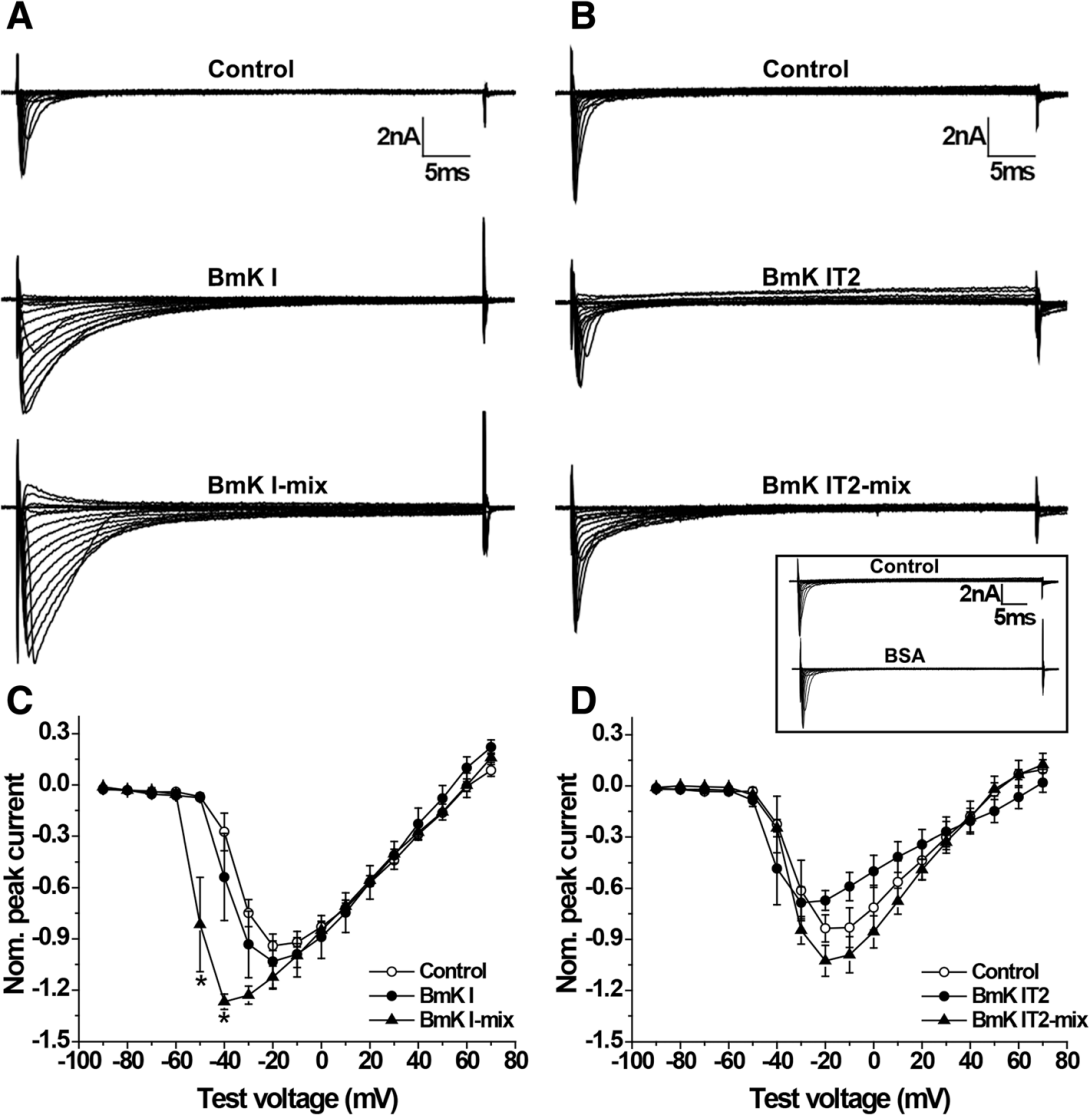
Effect of BmK I and BmK IT2 on DRG-type sodium channels. **a** Representative sodium currents under control conditions (top), after application of 400 nM BmK I (middle) and after application of 400 nM BmK I and 400 nM BmK IT2 to BmK I group (bottom), shown at intervals of 10 mV. **b** Representative sodium currents under control conditions (top), after application of 400 nM BmK IT2 (middle), and after application of 400 nM BmK I and 400 nM BmK IT2 to BmK IT2 group (bottom), shown at intervals of 10 mV. Representative sodium currents under control conditions, and after application of BSA (panel). **c** The I-V relationship of the VGSCs on ND7-23 cells in the absence (*n* = 6), presence of 400 nM BmK I (*n* = 5) and in presence of 400 nM BmK I and 400 BmK IT2 (*n* = 5). **d** The I-V relationship of the VGSCs on ND7-23 cells in the absence (*n* = 6), presence of 400nM BmK IT2 (*n* = 5) and in presence of 400 nM BmK I and 400 BmK IT2 (*n* = 5). *p < 0.05

The current was elicited by a 10 mV step from a holding potential of −120 mV during depolarization ranging from −90 to +70 mV. BmK I-mix group (BmK IT2-mix group) was defined as the ND7-23 cells that had been treated with the mixture of 400nM BmK I and 400nM BmK IT2 after pretreatement with 400nM BmK I (BmK IT2). The currents of sodium channels in ND7-23 were influenced after application of BMK I or BmK IT2 (Fig. 1a and b). BmK I-mix group and BmK IT2-mix group showed enhanced peak currents and slow inactivation, while BmK I-mix group showed entirely different currents compared to BmK IT2-mix group.

Current–voltage (I-V) curves in the absence or presence of BmK I or BmK I-mix are shown in Fig. 1c. In order to minimize diversity of different ND7-23 cells, all peak currents were normalized to the maximum peak current in control group. The normalized peak current was enhanced by over 20 % of control group in BmK I-mix group and by 10 % in BmK I group. The enhancement ratio of BmK I-mix group (I_NaP_ = −0.8158 ± 0.2764 at –50 mV; −1.268 ± 0.04475 at –40 mV, *n* = 5) was significantly larger than that of BmK I group (I_NaP_ = −0.07498 ± 0.007090 at –50 mV; −0.5382 ± 0.2540 at –40 mV, *n* = 5, *p* < 0.05) at –50 mV and –40 mV. Especially, the I-V curve of BmK I-mix group peaked at −40 mV. The minimum value was −1.268 ± 0.04475 (Fig. 1c, *n* = 5, *p* < 0.05). The I-V curve of controls and of BmK I peaked at −20 mV. The minimum value was −0.9405 ± 0.02835 (*n* = 6) and −1.032 ± 0.1610 (*n* = 5), respectively (Fig. 1c).

The ND7-23 cells were also treated with BmK IT2 and BmK I mixed with BmK IT2. The current–voltage (I-V) curves were also draw to count peak current, as shown in Fig. 1d. According to the report by Tan et al. [9], the normalized peak I_Na_ decreased to 0.8 fold of control (I_NaP_ = −0.8355 ± 0.07998, *n* = 6) in BmK IT2 group (Fig. 1d, I_NaP_ = −0.6714 ± 0.05813, *n* = 5, *p* < 0.05). Compared with the control group, the enhanced peak I_Na_ in BmK IT2-mix group (I_NaP_ = −1.026 ± 0.09113, *n* = 5) was not significantly different (Fig. 1d, *p* > 0.05).

### Voltage-dependent steady-state activation and inactivation were modulated by BmK I and BmK IT2

**Fig. 2:**
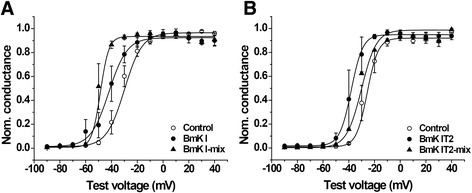
Effect of BmK I and BmK IT2 on the voltage dependence of steady-state activation. **a** The G-V curve of steady-state activation in the absence (*n* = 6), presence of 400 nM BmK I (*n* = 5) and in presence of 400 nM BmK I and 400 BmK IT2 (*n* = 5). **b** The G-V curve of steady-state activation in the absence (*n* = 6), presence of 400 nM BmK IT2 (*n* = 5) and in presence of 400 nM BmK I and 400 BmK IT2 (*n* = 5)

The peak of I-V curves in BmK I-mix group was more negative at hyperpolarized potentials, which was similar to the report described by Feng et al. [14]. In order to compute the relative value of conductance, we calculated the reversal potential. It was fitted to Boltzmann equation with its command voltage to draw the conductance-voltage (G-V) curve (Fig. 2a and b). The midpoint voltage (V_1/2-a_) and slope (K_a_) for activation were obtained from the G-V curve (Table 1). V_1/2-a_ of DRG-type sodium channels was significantly affected by post-treated BmK I and BmK IT2. As follows, the V_1/2-a_ was shifted to −48.55 ± 1.406 mV (*n* = 5) in BmK I-mix group from −29.99 ± 1.225 mV (*n* = 6) in control group (Fig. 2a, Table 1, *p* < 0.01), while BmK I shifted V_1/2-a_ to −42.02 ± 1.314 mV (Fig. 2a, Table 1, *n* = 5, *p* < 0.05). The K_a_ was did not show visible changes between control (K_a_ = 6.905 ± 0.5381, *n* = 6) and BmK I group (Table 1, Ka = 7.047 ± 0.5250, *n* = 5, *p* > 0.05), while K_a_ was obviously reduced in BmK I-mix group (Table 1, K_a_ = 3.288 ± 0.4903, *n* = 5, *p* < 0.05). The V_1/2-a_ of DRG-type sodium channels was shifted to −38.12 ± 0.9765 mV in BmK IT2 group (*n* = 5, *p* < 0.05), −30.14 ± 1.741 mV in BmK IT2-mix group (Table 1, *n* = 5, *p* > 0.05), and −25.68 ± 1.813 mV in control group (*n* = 6), respectively. The slope factors (K_a_) did not present obvious changes in BmK IT2 (K_a_ = 4.486 ± 0.3817, *n* = 5) and BmK IT2-mix group (K_a_ = 5.815 ± 0.7268, *n* = 5), compared with the control group (Fig. 2b, Table 1, *n* = 6, *p* > 0.05).

**Table 1:** Parameters for voltage dependence of steady-state activation and steady-state inactivation of the DRG-type sodium channels

	Steady-state activation		Steady-state inactivation		
	V_1/2-a_	K_a_	V_1/2-i_	K_i_	n
Control	−30.0 ± 1.22	6.90 ± 0.54	−71.9 ± 0.90	6.97 ± 0.66	6
BmK I	−42.0 ± 1.31*	7.05 ± 0.52	−75.6 ± 1.07	10.25 ± 0.64	5
BmK I-mix	−48.6 ± 1.40**	3.29 ± 0.49*	−76.8 ± 0.62	9.26 ± 0.33	5
Control	−25.7 ± 1.81	4.27 ± 0.59	−69.2 ± 1.12	7.78 ± 0.67	6
BmK IT2	−38.1 ± 0.98*	4.48 ± 0.38	−72.2 ± 0.86	7.91 ± 0.36	5
BmK IT2-mix	−30.1 ± 1.74	5.81 ± 0.73	−71.6 ± 1.06	9.84 ± 0.56	5

*p < 0.05, **p < 0.01, significant difference to control (one-way ANOVA, Tukey test)

**Fig. 3:**
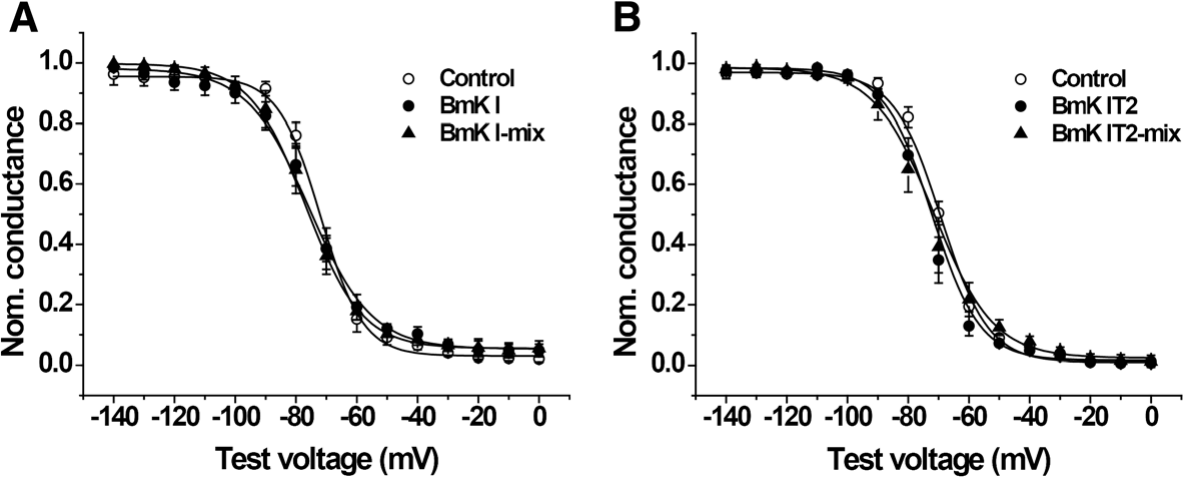
Effect of BmK I and BmK IT2 on the voltage dependence of steady-state inactivation. **a** The G-V curve of the steady-state inactivation in the absence (*n* = 6), presence of 400 nM BmK I (*n* = 5) and in presence of 400 nM BmK I and 400 BmK IT2 (*n* = 5). **b** The G-V curve of the steady-state inactivation in the absence (*n* = 6), presence of 400 nM BmK IT2 (*n* = 5) and in presence of 400 nM BmK I and 400 BmK IT2 (*n* = 5)

The voltage-dependent steady-state inactivation of DRG-type sodium channels was determined by eliciting 400-ms conditioning pulses to voltages between −140 and 0 mV in 10 mV increments followed by a 80 ms standard test pulse to 0 mV. The currents were also normalized and were calculated as relative conductance. Data were fit with a Boltzmann function to draw the conductance-voltage (G-V) curve (Fig. 3a and b). The half-inactivation voltage (V_1/2-i_) and the slope factor (K_i_) were calculated (Table 1). No significant difference was discovered in BmK I (*n* = 5, *p* > 0.05), BmK I-mix (*n* = 5, *p* > 0.05) compared to control (Fig. 3a, Table 1, *n* = 6), as well as the BmK IT2 (*n* = 5, *p* > 0.05), BmK IT2-mix (*n* = 5, *p* > 0.05) compared to control (Fig. 3b, Table 1, *n* = 6). Eventually co-applied BmK I and BmK IT2 did not modulate the steady-state inactivation of ND7-23 cells currents.

### Inhibition of open-state inactivation of DRG-type sodium channels

In order to confirm the modulative effects of BmK I and BmK IT2 on open-state inactivation, the inactivation phase of I_Na_ tracings was analyzed by fitting to single exponential function. The time constants from the peak value to the persistent current were calculated before and after applied BmK I and BmK IT2 at 10 mV to 30 mV.

**Fig. 4:**
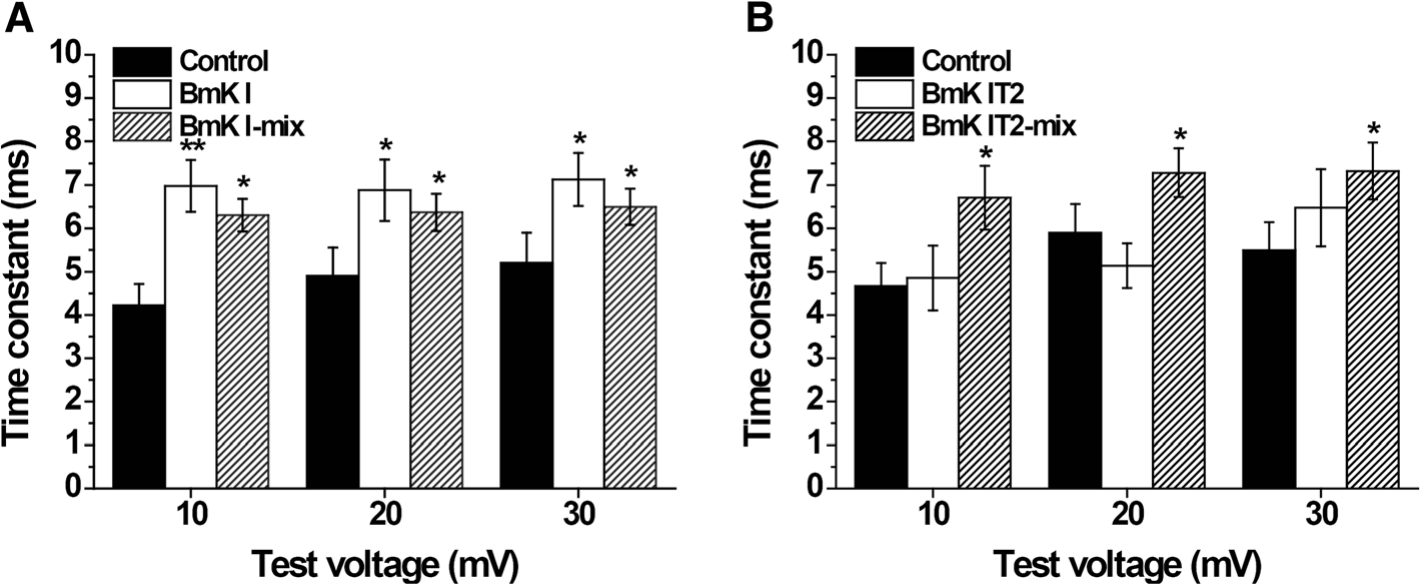
Effect of BmK I and BmK IT2 on inhibition of open-state inactivation of DRG type sodium channels. **a** The time constant of open-state inactivation in the absence (*n* = 10), presence of 400 nM BmK I (*n* = 10) and in presence of 400 nM BmK I and 400 BmK IT2 (*n* = 6). **b** The time constant of open-state inactivation in the absence (*n* = 9), presence of 400 nM BmK I (*n* = 5) and in presence of 400 nM BmK I and 400 BmK IT2 (*n* = 9)

The inactivation of sodium channels in ND7-23 was slowed down in BmK I group, BmK I-mix group and BmK IT2-mix group (Fig. 1a and b). When the ND7-23 cells were applied with BmK I, the time constant was significantly increased to 6.982 ± 0.5973 ms (*n* = 10, *p* < 0.01), 6.881 ± 0.7093 ms (*n* = 10, *p* < 0.05) and 7.132 ± 0.6140 ms (*n* = 10, *p* < 0.05) respectively at 10 mV, 20 mV and 30 mV while it was 4.223 ± 0.4944 ms (*n* = 10), 4.902 ± 0.6564 ms (*n* = 10) and 5.203 ± 0.6961 ms (*n* = 10) in control (Fig. 4a). In addition, the open-state inactivation of VGSCs in BmK I-mix group was still slower than in control group with the time constant of 6.306 ± 0.3762 ms (*n* = 6, *p* < 0.05), 6.371 ± 0.4289 ms (*n* = 6, *p* < 0.05) and 6.499 ± 0.4195 ms (*n* = 6, *p* < 0.05) at 10 mV, 20 mV and 30 mV (Fig. 4a). BmK IT2-mix group was shared an similar property, and the time constant was 6.710 ± 0.7331 ms (*n* = 9, *p* < 0.05), 7.284 ± 0.5559 ms (*n* = 9, *p* < 0.05) and 7.320 ± 0.6516 ms (*n* = 9, *p* < 0.05) in BmK IT2-mix group compared to 4.666 ± 0.5358 ms (*n* = 9), 5.899 ± 0.6654 ms (*n* = 9) and 5.499 ± 0.6426 ms (*n* = 9) in control group (Fig. 4b). BmK IT2 did not prolong the time constant of open-state inactivation, whose time constant was 4.854 ± 0.7474 ms (*n* = 5, *p* > 0.05), 5.138 ± 0.5182 ms (*n* = 5, *p* > 0.05) and 6.479 ± 0.8902 ms (*n* = 5, *p* > 0.05) (Fig. 4b).

## Discussion

In this study, the positive synergistic effect of BmK I and BmK IT2 on ND7-23 endogenous VGSCs was identified by whole cell patch clamp. From our results, we found out that BmK IT2 enhanced the pharmacological effect of BmK I by eliciting a higher peak current (Fig. 1c) and shifting the voltage-dependent activation to more hyperpolarized potential (Fig. 2a). The steady-state inactivation of VGSCs was not affected by co-applying BmK I and BmK IT2 (Fig. 3), which indicated that positive synergistic effect brought no extra effect on other pharmacological properties of sodium channels. It has been reported that the performance of site-3 toxins was enhanced by site-4 toxins in insects based on toxicity assays and binding experiments [15]. Our work provides another evidence of positive synergistic effect induced by two toxins on mammalian VGSCs by studying the modulation of gating properties, not the toxicity or binding experiments as previously done [15].

BmK I acts as a receptor site-3 modulator while BmK IT2 modulates sodium channels by binding to receptor site 4. It has been reported that the sensitivity of the receptor site 3 can be enhanced by specific ligands of another receptor site. For instance, brevetoxin (Pbtx-1) bound to receptor site-5 has positive allosteric modulation on LqhαITa site-3 toxin binding to insect sodium channels [16, 17]. In this case, we speculated that BmK IT2 seems to play a similar role as Pbtx-1. BmK IT2, binding to receptor site 4, may induce a conformational change on the channels. Moreover, the conformational change of channels not only modulates gating properties but also affect the binding of BmK I to receptor site 3. As was previously reported, receptor site 4 is mainly assigned to the S3-S4 in domain II, whereas site 3 involves S3-S4 in domain IV [18, 19]. The coupling interaction among voltage sensors of sodium channels has been reported, the positive synergistic effect of BmK I and BmK IT2 may be attributed to the allosteric interaction between receptor site 3 and site 4 [20–22].

It is worth noting that the peak current of sodium channels was not significantly enhanced in BmK IT2-mix group. The conductance-voltage curve in BmK IT2-mix group shifted less significantly than that in BmK IT2 group (Fig. 1d, Fig. 2b). The results suggested that BmK IT2 has two different sites for binding to DRG neuron-specific sodium channels. One was probably located in receptor site 4; the other was possibly located in receptor site 3. When the channels were treated with BmK I, BmK IT2 tended to bond to site 4. Moreover, BmK IT2 could bond to site 3 when the channels were pretreated with BmK IT2. BmK IT2 might share a similar binding property as BmK AS [23, 24].

Previous study showed that BmK I, the main lethal component of BmK venom, may induce various nociception behaviors in rats [8]. However, purified BmK I did not trigger higher pain levels in the same dosage of BmK venoms, though the pain response was dose-dependent [6, 8]. Besides, some other components, such as BmK IT2, performed anti-nociception effect in inflammatory pain model induced by formalin [10, 11]. Our results explained the severe pain induced by BmK venom and a novel perspective of scorpion sting induced pain. The non-toxic truncated β-toxins (ΔΔBj-xtrIT and ΔΔCss4) have been confirmed to be capable of modulating the binding and effects of site-3 scorpion α-toxins LqhαIT in an allosteric manner [25]. Similarly, both the toxicity and the content of BmK IT2 are much lower than that of BmK I. Considering that there are 100 to 300 peptides found in scorpion venom and most of them only have low toxicity. Our finding suggested a new functional role of non-lethal polypeptides in enhancing the effect of other active neurotoxins (the scorpion venom). This may be one of the reasons to explain why these non-lethal components have not disappeared over the course of animal evolution.

## Conclusions

The present results provide evidence that site-4 toxin (BmK IT2) could enhance the pharmacological effects of site-3 toxin (BmK I) on mammalian voltage-gated sodium channels. Meanwhile, this study declares that the allosteric interaction among different receptor sites play an important role in scorpion sting-induced pain.
